# The effect of mode and context on survey results: Analysis of data from the Health Survey for England 2006 and the Boost Survey for London

**DOI:** 10.1186/1471-2288-10-84

**Published:** 2010-09-27

**Authors:** Sarah Tipping, Steven Hope, Kevin Pickering, Bob Erens, Marilyn A Roth, Jennifer S Mindell

**Affiliations:** 1 National Centre for Social Research (NatCen), 35 Northampton Square, London EC1V 0AX, UK; 2 Department of Epidemiology & Public Health, UCL (University College London), 1-19 Torrington Place, London WC1E 6BT, UK

## Abstract

**Background:**

Health-related data at local level could be provided by supplementing national health surveys with local boosts. Self-completion surveys are less costly than interviews, enabling larger samples to be achieved for a given cost. However, even when the same questions are asked with the same wording, responses to survey questions may vary by mode of data collection. These measurement differences need to be investigated further.

**Methods:**

The Health Survey for England in London ('Core') and a London Boost survey ('Boost') used identical sampling strategies but different modes of data collection. Some data were collected by face-to-face interview in the Core and by self-completion in the Boost; other data were collected by self-completion questionnaire in both, but the context differed. Results were compared by mode of data collection using two approaches. The first examined differences in results that remained after adjusting the samples for differences in response. The second compared results after using propensity score matching to reduce any differences in sample composition.

**Results:**

There were no significant differences between the two samples for prevalence of some variables including long-term illness, limiting long-term illness, current rates of smoking, whether participants drank alcohol, and how often they usually drank. However, there were a number of differences, some quite large, between some key measures including: general health, GHQ12 score, portions of fruit and vegetables consumed, levels of physical activity, and, to a lesser extent, smoking consumption, the number of alcohol units reported consumed on the heaviest day of drinking in the last week and perceived social support (among women only).

**Conclusion:**

Survey mode and context can both affect the responses given. The effect is largest for complex question modules but was also seen for identical self-completion questions. Some data collected by interview and self-completion can be safely combined.

## Background

Local government and health agencies need robust, valid local level data on health and health-related behaviours. National surveys of the health of the general population are becoming more common [1] but these are not designed to provide data for local areas. Even a large survey like the Health Survey for England (HSE), an annual study of the health of the general population [2], would need an additional sample to enable local level analyses to be conducted. The optimal design for a boost sample is one which exactly matches that of the national survey. However, as most large scale national surveys in the UK use face-to-face interviewing, this may be prohibitively expensive for local boosts. Switching to a self-completion mode for the bulk of data collection maximises the sample size for a given budget, whilst retaining the features of the original sample design. Before making such a switch, measurement issues need to be taken into account. There is a large body of evidence to show that responses to survey questions may vary by mode of data collection [3,4]. This is due to the particular features of each mode, including the design and delivery of the survey instrument, and the 'actors' involved in the survey process, namely respondents and interviewers [5]. For example, the presence of an interviewer may improve data quality by guiding the respondent through a complex questionnaire [6], but a self-completion mode may encourage respondents to give more honest answers to sensitive questions [7,8]. When considering combining data from different modes it is therefore important to assess issues of comparability [9].

The London Health Observatory (LHO) led a pan-London consortium to commission a local boost to the HSE using the less costly method of self-completion questionnaires. The Boost Survey for London used the same sampling strategy as the HSE but a different mode of data collection. Comparisons of the two samples showed few significant differences between the socio-demographic characteristics of the achieved households and individual adults. Moreover, after non-response weights were applied, both samples provide reasonably close correspondence with the London population for the characteristics examined [10]. This paper examines the extent to which the data from London participants in the HSE 2006, which primarily used face-to-face interviews, and the Boost Survey for London, which used self-completion questionnaires, are comparable, and the size and direction of any differences. The results for a number of health and health-related behaviours are compared.

## Methods

### Data and participants

The HSE is an cross-sectional survey that uses a new random sample each year. The core HSE sample is designed to be representative of the population in England living in private households. Data are collected about households and individuals using computer-assisted personal interviewing (CAPI). The interviewer also measures the participants' height and weight. A small amount of information is collected during the interview using self-completion questionnaires [10-12]. Participants are later visited separately by a nurse for further measurements and the collection of biological samples. 58% of eligible households completed a household interview. 85% of households members aged 16 and over gave an individual interview, giving 1,569 interviews in total [12].

The Boost Survey for London used self-completion questionnaires rather than face-to-face CAPI. Selected households were visited by interviewers, who conducted a short household interview (using a paper questionnaire), and distributed paper questionnaires to household members. Interviewers later returned to households to collect completed individual questionnaires and to encourage non-responding individuals to participate. Interviewers also measured the height and weight of all consenting participants who were present at either of the visits. There was no nurse visit. Questions were taken from the HSE self-completion questionnaires where these existed. Otherwise HSE questions were converted to self-completion format with the aim of keeping the question wording as similar as possible to the HSE interview. 61% of eligible households and 65% of individuals in responding households aged 16 and over completed an interview, giving 5,004 interviews in total.

The samples for both surveys were selected using the same method, described in detail elsewhere [10,11]. All addresses were selected from the Royal Mail's Postcode Address File (PAF). The sample was drawn in two stages; at the first stage a sample of postcode sectors were drawn as the primary sampling units (PSUs), with probability proportional to the total number of addresses within them. 102 London PSUs were selected for the HSE core sample and 202 for the London Boost. At the second stage 20 addresses were selected from each HSE PSU. For the London Boost, 40 addresses were selected within each inner London PSU, where response rates were expected to be lower, and 34 addresses were selected per outer London PSU. Prior to selection, the PAF was stratified by local authority (PCT within London) and the percentage of households with a head of household in a non-manual occupation (Socio-Economic Groups 1-6, 13), taken from the 2001 Census. This was done to increase the precision of the sample.

All participants aged 16 years or over from the Boost Survey for London and all HSE participants of that age who were part of the core sample and resident in one of London's Primary Care Trusts were included in the analyses. Ethical approval was obtained for both surveys from the London Multi-centre Research Ethics Committee prior to the surveys starting. Throughout the remainder of this paper we refer to the London residents of the national HSE Core sample as the 'Core' and participants in the Boost Survey of London as the 'Boost'.

Whilst each questionnaire covered the same topics, the mode of delivering questions could differ. Most questions on the Core survey were asked by an interviewer using a CAPI questionnaire, whereas Boost participants completed a paper questionnaire. For some sets of questions, this affected the ease of finding the next question.

To elicit information on fruit and vegetable consumption and on physical activity, questions on the Core survey were asked by an interviewer using a Computer Aided Personal Interviewing (CAPI) questionnaire enabled automatic question routing. However, Boost participants completed the paper questionnaire so had to follow some complex routing themselves.

Participants were asked about their fruit and vegetable consumption on the previous day, used to calculate the number of portions of fruit and vegetables eaten that day. When generating the summary measure, portions of fruit juice, dried fruit and pulses were capped at one portion each, regardless of how many were actually consumed, in accordance with Food Standards Agency guidelines [4].

Questions on physical activity were used to measure the proportion of participants meeting the government's recommendation of 30 minutes or more of at least moderate intensity physical activity on five or more days a week. Both surveys asked about participation in heavy housework, manual/gardening/DIY ('Do-It-Yourself'), walking, sports and occupational activity. Questions ascertained the number of days and the average amount of time spent doing each activity, and whether the activity caused them to become out of breath or sweaty.

In the Core face-to-face interview, participants were presented with a show card listing 10 different activities, which were also read aloud by the interviewer. The amount of filtering used in the CAPI was much too complex for a self-completion format, so Boost participants were provided with a list of the same 10 activities (and space to add up to three extra). The list included boxes for participants to record the number of days and time spent in each activity in the last four weeks, and whether or not the activity made them out of breath or sweaty.

Participants were asked to rate their general health as very good, good, fair, bad or very bad. They were then asked if they had any longstanding illness, and if so, whether it limited their daily activities. Although asked in different modes, these questions used identical wording in both the self-completion and face-to-face surveys. Some questions were asked in the same mode. Questions about perceived social support the General Health Questionnaire (GHQ) 12, an indicator of psychological health, in which twelve questions are used to assess the participant's present state relative to their usual, or normal, state [13], were both asked in a self-completion format.

Smoking and alcohol use were collected via CAPI for the Core aged 25 and over and via a self-completion questionnaire for Core participants aged 16-17 interviewers used their discretion to choose which mode to use for Core adults aged 18-24 years. All Boost participants were asked the questions in the self-completion format. Thus the context of the data collection varied: the presence or absence of an interviewer when the questions were answered, which other questions were in the self-completion questionnaire, and the order in which the topics were covered.

### Analysis

Two complementary approaches were used to examine whether there were differences by data collection mode for estimates of health and health-related behaviour. A comparison was made of weighted estimates taken from the two surveys to seek evidence of any residual bias that remained after non-response weights had been applied. These weights correct for unequal selection probabilities and the effects of differential non-response. The two samples were weighted separately using the same weighting procedure, which corrected for differential responses by age, sex, household type and inner/outer London. More details are given in the Additional file 1 and elsewhere [11]. These weighted estimates are the figures that would be reported from each survey.

The comparisons were then run a second time: non-response weights were not applied but instead an adjustment was made to reduce the differences in sample composition. If a difference were identified by both methods, then it was more likely to be a genuine effect of survey mode. The socio-demographic profile of the achieved Core sample was adjusted to match the achieved Boost sample using propensity score matching (PSM) [14]. PSM is a method that allows cases from a treatment sample (in this case the HSE Core sample in London) to be matched to cases from a control sample (the London Boost Sample). The matching controls for differences in sample profile; in this case the socio-demographic profile of the Core sample is adjusted to make it match that of the Boost. Matching the samples means any differences in survey estimates should be attributable to measurement error and not sample composition[13]. Further details are provided in the Additional file 1 and elsewhere [11]. 

The comparison of key results was then carried out on the matched samples, so that any differences could be attributable to measurement error rather than sample composition. The analysis used the same methods to examine the effect of differential response but the matched weight was applied rather than the traditional non-response weight. The Core sample was adjusted to match the *unweighted *Boost sample, so the samples would be comparable with one another. For the remainder of this paper this is referred to as "matched data".

Although non-response weighting adjusts the samples to the same population and makes them similar in terms of age and sex profiles, PSM results in two samples that are a closer match because a wider pool of characteristics can be used to match the two samples.

The analyses were carried out using chi-square tests and two-tailed t-tests. Simple statistical tests are appropriate rather than more complex analyses because the matching controls for differences in the same way as a regression model. For each variable, the probability of the difference occurring by chance had the two samples come from the same population was calculated.

As both samples were clustered, stratified and weighted, the analysis was run using the 'svy' commands in Stata 10 to account for the complex sample design. Men and women were analysed separately to minimise clustering at the household level.

Item non-response was higher in the Boost than Core survey [10,11]. The survey estimates in this report are based on valid estimates only; no attempt has been made to impute missing data. For each comparison the missing responses were excluded.

All figures in the text are based on weighted data. Unless otherwise stated, the patterns in the matched data were the same as those found in the weighted data.

## Results

There were no significant differences in results between the Core and Boost samples for a number of variables including: long-term illness, limiting long-term illness, current rates of smoking, whether participants drank alcohol and how often they usually drank (Table 1). There were differences, however, in levels of consumption reported. Women smokers in the Boost sample reported smoking on average 12.8 cigarettes per day, whereas the weighted mean for Core participants was 10.1 (p < 0.05). This difference was not significant in men (mean 13.5 for the Core and 12.4 for the Boost, weighted data).

**Table 1:** Results not affected by survey mode

	WOMEN		MEN	
	**Core**	**Boost**	**Core**	**Boost**

	**%**	**%**	**%**	**%**

**Longstanding illness (%)**				

None	69	67	72	70

Non-limiting	13	11	11	12

Limiting	19	21	17	19

*p*		*0.23*		*0.53*

N	833	2676	736	2174

**Smoking status (%)**				

Current smoker	19	19	25	25

Ex-smoker	23	22	28	25

Never smoked	58	60	47	50

*p*		*0.57*		*0.28*

N	827	2680	728	2142

**No. of smokers in household**				

None	76	77	76	76

One	16	15	16	16

Two	7	8	8	9

*p*		*0.75*		*0.93*

N	833	2736	783	2206

**Frequency of alcohol use in past 12 months**				

Almost every day	9	6	15	14

5-6 d pw	4	4	8	7

3-4 d pw	11	13	16	16

Once or twice a week	21	22	27	25

Once or twice a month	12	12	10	11

Once every couple of months	9	9	3	6

Once or twice a year	8	10	5	5

Not at all/non-drinker	26	24	16	16

*p*		*0.19*		*0.43*

N	731	2297	644	1918

**Whether drank in the last 7 days^a^**				

Yes	68	66	80	78

No	32	34	20	22

*p*		*0.33*		*0.29*

N	535	1814	539	1635

**^a ^**Excluding those who did not drink in the last 12 months

Similarly, there were differences in the number of units of alcohol recorded, with Boost participants reporting a greater number of units than Core participants (Table 2, showing weighted and match data) and having a higher prevalence of binge drinking. Of the participants over the age of 25, 44% of women and 50% of men in the Boost sample who had drunk alcohol in the previous week had exceeded the thresholds for binge drinking, compared with 33% of women and 34% of men in the Core.

**Table 2:** Alcohol consumption on the heaviest drinking day in the last week^a^

	WOMEN			MEN		
	Core	Boost	*P-value*	Core	Boost	*P-value*
**Mean units of alcohol**	Units	Units		Units	Units	
Weighted	4.7	6.0	*0.000*	7.1	8.8	*0.000*
Matched	4.6	5.9	*0.000*	6.5	8.6	*0.000*
**Proportion binge-drinking ^b^**	%	%		%	%	
Weighted	33	44	*0.003*	34	50	*0.000*
Matched	33	42	*0.005*	31	48	*0.000*
N	358	1079		425	1167	

^a ^All participants aged 25 and over who drank in the last week

^b ^Drinking more than double the recommended daily limit (i.e. >8 units in men, >6 units in women).

There were large differences in the responses regarding general health. The difference in the distribution of responses was highly significant (p < 0.001) for both men and women, with Core participants much more likely to say that their health was very good. Boost participant answers were more likely to be in the middle of the distribution and to state their general health was fair (Table 3).

**Table 3:** Results affected by survey mode: general health and fruit and vegetable consumption^a^

	WOMEN		MEN	
	**Core**	**Boost**	**Core**	**Boost**

	**%**	**%**	**%**	**%**

**Self-assessed general health**				

Very good	33	21	36	24

Good	45	51	44	48

Fair	14	23	14	24

Bad	6	4	5	3

Very bad	2	1	1	1

*P-value*		*0.000*		*0.000*

Very good/good	78	72	79	72

Fair/bad/very bad	22	28	21	28

*P-value*		*0.000*		*0.000*

N	833	2701	736	2181

**Portions of fruit and vegetables**				

Less than 1	5	4	8	6

1 to 2	26	15	26	17

3 to 4	27	27	28	24

5 or more	42	55	38	54

*P-value*		*0.000*		*0.000*

N	833	2647	735	2133

**^a ^**results shown for weighted data

The proportion of participants with a GHQ12 score of zero was higher for Core participants than Boost participants; Boost participants were more likely to have a score of four or more, which indicates poor psychological health. Similarly, women in the Boost were significantly more likely than women in the Core to report either a severe lack, or no lack, of social support, although the actual percentage differences were not large. The same was not found for men (Table 4, showing matched data).

**Table 4:** Results differing between surveys: GHQ12 and Perceived social support^a^

	WOMEN		MEN	
	**Core**	**Boost**	**Core**	**Boost**

	**%**	**%**	**%**	**%**

**GHQ12 score - grouped**				

Score 0	58	51	63	57

Score 1-3	27	28	24	27

Score 4+	15	21	12	16

*P-value*		*0.005*		*0.011*

N	691	2628	620	2143

**Perceived social support score**				

No lack	62	64	55	59

Some lack	25	20	24	21

Severe lack	13	16	22	20

*P-value*		*0.036*		*0.223*

N	701	2665	626	2133

**^a ^**results shown for matched data

Note: These questions were asked in the same mode

Boost participants reported consuming more portions of fruit and vegetables than Core participants. The differences between sample types were large:; 54% of men and 55% of women in the Boost sample met the government's 5-a-day recommendation, compared with 38% of men and 42% of women in the Core (Table 3).

The number of portions recorded by Boost participants for each category of fruit, juice, vegetable and salad was consistently higher than the number recorded by Core participants. Boost participants were more likely to say they had eaten each category of fruit and vegetable listed and, where a particular category had been recorded, they generally entered a greater number of portions.

There were also large differences between the Core and Boost samples in reported physical activity. The summary physical activity level for Boost participants was much lower than for Core participants (Table 5), despite the reported rates of participation in sports and other activities being generally higher in the Boost sample. These differences are likely to be caused by the amount of missing data in the Boost, as described elsewhere [10,11]. Since the individual questions were combined to derive the summary activity measure, missing just one of the component variables resulted in a missing value for the summary variable. The summary physical activity measure was missing for 16% of Boost participants, compared with 0.2% of Core participants.

**Table 5:** Summary physical activity level for participants aged 16-64^a b^

	WOMEN		MEN	
	Core	Boost	Core	Boost
**Summary physical activity level**	**%**	**%**	**%**	**%**
Meets recommendations	34	18	44	22
Some activity	34	39	28	35
Low activity level	32	43	28	43
*P-value*		*0.000*		*0.000*
N ^a^	695	1957	620	1593

^a ^The analysis includes participants aged 16-64 only, since the physical activity module was not asked of all older participants in the Core survey.

^b ^results shown for weighted data

## Discussion

There were no significant differences between the Core and Boost for smoking prevalence, frequency of alcohol consumption, longstanding illness and limiting longstanding illness, all questions with simple answer categories. However, significant differences were found between the Boost and the Core for a number of other variables.

The Boost data showed significantly higher estimates of the number of cigarettes smoked by women and the number of alcohol units drunk by both men and women. If it is assumed that higher levels of reporting are due to respondents being more honest, then these results are consistent with other studies which have shown that self-completion questionnaires are more likely than face-to-face interviews to elicit honest responses about potentially sensitive behaviours such as levels of smoking and drinking [15-17].

Results from the fruit and vegetable consumption and physical activity question modules varied considerably between the Core and Boost surveys. These appear to be due to key differences in question format and mode of delivery. It could be that a CAPI format encourages under-reporting once it becomes clear to Core participants that a positive response to an initial question leads to an extra set of follow-up questions. Moreover, both the physical activity and fruit and vegetable consumption modules are cognitively demanding and require much attention to detail. Both the complexity of the filtering and the unavailability of the interviewer to provide guidance are likely to have influenced the results for Boost participants and certainly led to higher levels of missing data.

Without an interviewer being present to provide guidance, it may be that Boost respondents over-estimate their participation in physical activities by including sports and other activities that fall outside the four week period being asked about. Similarly, their levels of fruit and vegetable consumption is likely to be higher as there is a greater temptation in the self-completion format to include consumption that falls outside the one-day period covered in the questionnaire.

The complexity of the filtering for these modules meant that the self-completion format used for the Boost results in much higher levels of missing data, so that when complex variables are derived combining responses to many questions, the level of missing information is much higher for Boost than Core respondents (as there is very little missing data in a CAPI interview). Moreover, the chances to have missing data increase with increasing levels of participation or consumption. This probably explains the apparent paradox that Boost respondents were more likely to report participation in physical activities, but had a lower overall level of physical activity when combining the questions that make up that variable. By reporting more activities, the additional questions requiring an answer also increase, thereby increasing the chance of missing a question, which could result in the exclusion of the more active individuals from the summary variable for the Boost respondents.

The difference in results for self-reported general health was surprising as Core and Boost participants answered identically phrased questions. However, there is evidence that the visual presentation of a question gives meaning over and above the content of the question itself [18]. Visualising the scale on paper may have influenced participants in the Boost to choose the less extreme categories. Alternatively, Core participants may have been more likely to give a socially desirable answer (i.e. very good) because of the presence of an interviewer. There is some evidence to suggest respondents are more likely to give positive ratings of their health when an interviewer is present, compared to a self-completion mode [7]. Leading on from this, it is possible that the presence of other household members during the interview could also have an impact on the responses given.

However, further investigation of the Core sample showed no differences in the responses between those interviewed alone and those interviewed concurrently with another household member. This suggests that there are particular effects on response caused by an interviewer's presence, rather than the presence of other household members. In addition, it may be the appearance of the scale on paper, versus having it read out, that results in the difference. Including the scale on a show card during an interview may therefore mitigate the difference found between modes.

Finally, the GHQ12 and social support modules were asked in identical self-completion format in both the Core and Boost surveys, yet had different results, though these were small for social support. The differences may have been caused by the context in which the questionnaire was delivered. The presence of other household members was found not to affect the Core responses, however, the presence of an interviewer may have affected how Core participants answered, even though the interviewer was not directly asking the questions. The placement of these questions may also have affected the responses: the GQH12 questions were near the beginning of the Boost questionnaire, whereas the self-completion booklet was administered near the end of the Core interview, putting them in a slightly different context.

### Strengths and limitations of this study

The main strengths of this study were the use of the same sampling frame, geographic area and method for drawing the samples and recruiting the participants. The household and individual response rates differed between the two surveys, in addition, the impact of non-response on the sample could differ by mode [10]. However, this study attempted to remove any differences in sample composition caused by different rates of participation through the use of non-response weighting and sought to validate the findings by repeating the analysis on samples matched for socio-demographic characteristics.

It is also possible that some non-response bias remained after non-response weights were applied. Some differences, such as a preference by respondents for a particular mode, cannot be measured and corrected for by weighting. It is also possible that some differences between the two samples remained after the matching process. However, the majority of estimates that were significantly different in the weighted data were also different in the matched data. This suggests that the larger differences in the survey estimates were not due to differences in sample composition or differential non-response but due to differences in measurement error.

For measures where the estimates differed significantly between the two surveys, it can be difficult to determine how much of the difference was caused by data collection mode and how much is due to other effects. It is also difficult to determine which estimate is nearer the 'true' population value. The literature suggest that self-completion data should be more accurate for sensitive or 'anti-social' behaviours [7]. However, it is unclear which mode of data collection elicits more accurate responses for questions about self-assessed general health and GHQ12 score.

## Conclusion

We have shown that data from some items collected using face-to-face interview and self-completion modes can be safely combined, for example prevalence of smoking and frequency of alcohol consumption. But we have also found differences between surveys for other items which are likely to reflect mode-specific factors, both in terms of questionnaire design and the effect of the presence or absence of an interviewer. Collection of complex data using self-completion questionnaires that attempt to parallel CAPI interview modules for topics such as fruit and vegetable consumption or levels of physical activity participation present many problems - in particular to do with the routing - and is not recommended unless the modules are re-designed (and thoroughly tested) to work in a self-completion format [19]. The use of a mixed mode strategy requires careful consideration of comparability between modes. Further experimental work with randomised assignment to mode should be carried out to investigate measurement issues in more detail. In addition, projects planning to use a mixed-mode approach should undertake thorough pre-testing of equivalence between the instruments used in each mode.

## Competing interests

All authors are involved in various aspects of conducting surveys as part of their employment.

## Authors' contributions

MR analyzed the data and noticed the discrepancies; JM conceived this study; BE and KP designed the analyses; JM and BE co-ordinated the study; ST, KP and SH conducted the analyses; ST, MR and JM drafted the initial manuscript. All authors contributed to interpreting the results, revising the manuscript, and read and approved the final manuscript.

## Pre-publication history

The pre-publication history for this paper can be accessed here:

http://www.biomedcentral.com/1471-2288/10/84/prepub

## Supplementary Material

Additional file 1 **Appendix: Non-response weighting and propensity score matching**. The supplementary file provides additional material about the methods used to generate non-response weights and more details on propensity score matching. This information is provided for those wishing to know more about the method or to see the detailed results of the regression for men and women to produce the weights used in the matching. Click here for file
