# Supplementary material for: The effect of mode and context on survey results: Analysis of data from the Health Survey for England 2006 and the Boost Survey for London
Source: BMC Med Res Methodol. 2010 Sep 27;10:84. doi: 10.1186/1471-2288-10-84 (PMC2955657; doi:10.1186/1471-2288-10-84)
Supplement: Additional file 1 — Appendix: Non-response weighting and propensity score matching. The supplementary file provides additional material about the methods used to generate non-response weights and more details on propensity score matching. This information is provided for those wishing to know more about the method or to see the detailed results of the regression for men and women to produce the weights used in the matching. [file 1471-2288-10-84-S1.DOC]

##### ADDITIONAL FILE

Further information on non-response weighting and propensity score matching

##### Non-response weighting

The selection weights correct for the effects of over-sampling addresses in smaller PCTs in the London Boost and the selection of households at multi-household addresses. The selection weights ensure the distribution of Boost addresses is correct across PCTs. By applying the selection weights it is possible to make assertions about the differences in the size of unweighted estimates as though the two samples had both been drawn with equal probability.

The non-response weights correct for the effects of differential non-response. The same methodology was used to create non-response weights for each sample, which is described in more detail below. These weights allow a comparison of key estimates that have been corrected for both unequal selection and non-response. The different stages of the weighting are described in more detail below.

**Address selection weight (w_0_)**

The Boost sample was designed to give an equal number of interviews per PCT, with an additional boost in Camden and Islington. Six Primary Sampling Units (PSUs [[1]](#footnote-2)) were selected from each London PCT, except Camden and Islington, where 14 PSUs were selected in each PCT. In addition, a larger number of addresses were selected from inner London PCTs, as the response rates in inner London were expected to be lower. There were 40 addresses selected in each inner London PCT, compared with 34 in outer London.

Address selection weights are needed for the Boost sample as the sampling probabilities for Boost addresses vary by PCT. The smaller PCTs have larger selection probabilities as the addresses within them were being sampled at a higher rate. Without selection weights the smaller PCTs would be over-represented in the sample. The Core sample does not require selection weights since each Core address had an equal chance of being selected.

The address selection weight (w_0_) was generated as the inverse of the address and PSU selection probabilities. This was equal to 1 for the Core sample, since the addresses in the Core were selected with equal probability.

**Household selection weight (w_1_)**

A small number of addresses (<1%) selected from the PAF contain more than one household. At these addresses the interviewers carry out a selection procedure to identify which households to include in the sample. The same procedure was used for both samples; interviewers selected up to three households at each address; if more than three households were found the interviewer selected three at random.

The household selection weight (w_1_) corrects for this selection of households and prevents households in multi-occupied addresses from being under-represented in the issued sample. This weight is equal to the number of households identified divided by the number selected. This weight was trimmed at 2 to avoid any large values.

The final selection weights were the product of the household and address selection weights (w_01_). The weights were scaled to match the achieved sample size. This weight (selectin) is used for the comparison of key estimates before corrections were made for differential non-response.

**Calibration weight for participating households (w_2_)**

Calibration weighting was used to generate weights for the participating households using CALMAR. The same variables were used for both Boost and Core samples. The achieved household sample was calibrated so that the distributions for age/sex and region for the household members matched the adjusted ONS 2005 mid-year household population estimates[[2]](#footnote-3) (Tables A1 and A2). The region variable used was an inner/outer London split based on PCT. The calibration weight generated for a particular household depended upon the age/sex profiles of all household members and the region within which it was situated. Since this is a household-level weight, information on all household members was used, including children. The household and address selection weights were used as initial values when generating the calibration weights (w_2_).

Table A1 ONS mid-2005 household population estimates for London by age and sex

| **Age (grouped)** | **Men** | **Women** |
| --- | --- | --- |
|  |  |  |
| 0-4 | 261,377 | 251,084 |
| 5-10 | 260,458 | 252,762 |
| 11-15 | 214,191 | 203,149 |
| 16-24 | 430,969 | 430,607 |
| 25-34 | 734,464 | 722,543 |
| 35-44 | 650,592 | 616,758 |
| 45-54 | 430,424 | 444,336 |
| 55-64 | 314,280 | 342,595 |
| 65-74 | 210,890 | 239,429 |
| 75+ | 160,099 | 242,261 |
|  |  |  |
| All London | 3,667,744 | 3,745,524 |

Table A2 ONS mid-2005 household population estimates for inner/outer London PCTs

| **Region** | **Estimate** |
| --- | --- |
|  |  |
| Inner London^1^ | 2,920,805 |
| Outer London | 4,492,463 |
|  |  |
| All London | 7,413,268 |

^1^Inner London PCT codes are: 5C3, 5C4, 5C5, 5C9, 5H1, 5K7, 5K8, 5LA, 5LC, 5LD, 5LE, 5LF and 5LG. Outer London PCT codes are: 5A4, 5A5, 5A7, 5A8, 5A9, 5AT, 5C1, 5C2, 5HX, 5HY, 5K5, 5K6, 5K9, 5M6, 5M7, 5NA, 5NC and TAK.

The aim of the calibration weighting was to reduce non-response bias resulting from differential non-response at the household level. The calibration weights generated (w_2_) were re-scaled so that the sum of the weights equalled the number of participating households. This weight is the household weight for the Core sample (hhwt).

**Adult interview weight (w_3_)**

There were no selection weights for adult respondents since all adults in responding households were selected for the Core sample. Non-response weights were generated to reduce possible non-response bias caused by individuals in responding households not completing individual interviews. Response was lower for the Boost sample; for the Boost 66% of adults in households with more than one adult completed an individual interview, compared with 86% for the Core.

A weighted (by hhwt) logistic regression model was fitted. The outcome was whether the interview was completed or not. The following variables were entered as covariates:

- Age group by sex,
- Household type, and
- Inner/outer London

The logistic regression model shows the relationship of these measures with response (see Tables A3 & A4). The adult non-response weight (w_3_) was calculated as the inverse of the predicted probabilities of response estimated from the regression model. Respondents in single adult households were not included in the modelling and were given a non-response weight of 1.

The weights were trimmed at the 0.5% tails to remove extreme values. The interview weights for the core sample were calculated as:

intwt = w_2_ x w_3_

The weights were re-scaled so that the sum of the weights equalled the size of the achieved sample.

Table A3 Adult individual non-response model for Core HSE data

|  | **B** | **S.E.** | **Wald** | **df** | **Sig.** | **Exp(B)** |
| --- | --- | --- | --- | --- | --- | --- |
|  |  |  |  |  |  |  |
| Age group |  |  | 25.82 | 13 | 0.02 |  |
| Men 16-24 |  |  |  |  | (baseline) |  |
| Men 25-34 | 0.28 | 0.27 | 1.04 | 1 | 0.31 | 1.32 |
| Men 35-44 | 0.28 | 0.29 | 0.88 | 1 | 0.35 | 1.32 |
| Men 45-54 | -0.06 | 0.30 | 0.04 | 1 | 0.85 | 0.94 |
| Men 55-64 | 1.14 | 0.45 | 6.40 | 1 | 0.01 | 3.12 |
| Men 65-74 | 0.94 | 0.55 | 2.99 | 1 | 0.08 | 2.57 |
| Men 75+ | 1.72 | 0.87 | 3.96 | 1 | 0.05 | 5.60 |
| Women 16-24 | 0.27 | 0.29 | 0.88 | 1 | 0.35 | 1.31 |
| Women 25-34 | 0.36 | 0.28 | 1.68 | 1 | 0.20 | 1.44 |
| Women 35-44 | 0.95 | 0.35 | 7.46 | 1 | 0.01 | 2.57 |
| Women 45-54 | 1.05 | 0.34 | 9.51 | 1 | 0.00 | 2.86 |
| Women 55-64 | 0.46 | 0.38 | 1.44 | 1 | 0.23 | 1.58 |
| Women 65-74 | 0.31 | 0.44 | 0.49 | 1 | 0.48 | 1.36 |
| Women 75+ | 0.99 | 0.67 | 2.17 | 1 | 0.14 | 2.70 |
|  |  |  |  |  |  |  |
| Household type |  |  | 44.31 | 4 | 0.00 |  |
| 2 adults, both 16-59,  no children | | |  |  | (baseline) |  |
| Small family | 0.39 | 0.25 | 2.56 | 1 | 0.11 | 1.48 |
| Large family | 0.38 | 0.32 | 1.40 | 1 | 0.24 | 1.47 |
| Large adult household | -0.76 | 0.18 | 17.01 | 1 | 0.00 | 0.47 |
| 2 adults, 1 or both aged 60+, no children | 0.17 | 0.35 | 0.24 | 1 | 0.62 | 1.19 |
|  |  |  |  |  |  |  |
| Region |  |  | 3.24 | 1 | 0.07 |  |
| Inner London | |  |  |  | (baseline) |  |
| Outer London | 0.25 | 0.14 | 3.24 | 1 | 0.07 | 1.29 |
|  |  |  |  |  |  |  |
| Constant | 1.17 | 0.26 | 19.44 | 1 | 0.00 | 3.21 |

Notes: 1. The response is 1 = response, 0 = non-response.

2. The model R^2^ = 0.062 (Cox and Snells).

3. B is the estimate coefficient with standard error S.E.

4. The Wald-test measures the impact of the categorical variable on the model with the appropriate number of degrees of freedom df. If the test is significant (sig < 0.05) then the categorical variable is considered to be ‘significantly associated’ with the response variable and therefore included in the model.

5. The Wald test for each level of the categorical variable is also shown. This tests the difference between that level and the baseline category.

Table A4 Adult individual non-response model for London Boost data

|  | **B** | | **S.E.** | **Wald** | **df** | **Sig.** | **Exp(B)** |
| --- | --- | --- | --- | --- | --- | --- | --- |
|  |  | |  |  |  |  |  |
| Age group |  | |  | 85.4 | 13 | 0.00 |  |
| Men 16-24 |  | |  |  |  | (baseline) |  |
| Men 25-34 | 0.15 | | 0.11 | 1.8 | 1 | 0.18 | 1.17 |
| Men 35-44 | 0.31 | | 0.12 | 6.8 | 1 | 0.01 | 1.37 |
| Men 45-54 | 0.74 | | 0.13 | 30.4 | 1 | 0.00 | 2.10 |
| Men 55-64 | 0.36 | | 0.16 | 5.2 | 1 | 0.02 | 1.43 |
| Men 65-74 | 0.07 | | 0.19 | 0.1 | 1 | 0.70 | 1.08 |
| Men 75+ | -0.36 | | 0.25 | 2.1 | 1 | 0.15 | 0.70 |
| Women 16-24 | 0.34 | | 0.12 | 7.4 | 1 | 0.01 | 1.40 |
| Women 25-34 | 0.27 | | 0.12 | 5.3 | 1 | 0.02 | 1.30 |
| Women 35-44 | 0.67 | | 0.12 | 29.0 | 1 | 0.00 | 1.96 |
| Women 45-54 | 0.67 | | 0.13 | 26.5 | 1 | 0.00 | 1.95 |
| Women 55-64 | 0.33 | | 0.15 | 4.5 | 1 | 0.03 | 1.39 |
| Women 65-74 | -0.13 | | 0.21 | 0.4 | 1 | 0.54 | 0.88 |
| Women 75+ | -0.35 | | 0.26 | 1.9 | 1 | 0.17 | 0.70 |
|  |  | |  |  |  |  |  |
| Household type |  | |  | 30.9 | 4 | 0.00 |  |
| 2 adults, both 16-59, no children |  | | |  |  | (baseline) |  |
| Small family | 0.64 | | 0.08 | 57.6 | 1 | 0.00 | 1.90 |
| Large family | 0.30 | | 0.11 | 7.7 | 1 | 0.01 | 1.35 |
| Large adult household | -0.06 | | 0.07 | 0.8 | 1 | 0.38 | 0.94 |
| 2 adults, 1 or both aged 60+, no children | 0.19 | | 0.17 | 21.2 | 1 | 0.00 | 1.21 |
|  |  | |  |  |  |  |  |
| Region |  | |  | 5.8 | 1 | 0.02 |  |
| Inner London | | |  |  |  | (baseline) |  |
| Outer London | | 0.13 | 0.06 | 5.8 | 1 | 0.02 | 1.14 |
|  | |  |  |  |  |  |  |
| Constant | | -0.13 | 0.11 | 1.6 | 1 | 0.21 | 0.87 |

Notes: 1. The response is 1 = response, 0 = non-response.

2. The model R^2^ = 0.084 (Cox and Snells).

3. B is the estimate coefficient with standard error S.E.

4. The Wald-test measures the impact of the categorical variable on the model with the appropriate number of degrees of freedom df. If the test is significant (sig < 0.05) then the categorical variable is considered to be ‘significantly associated’ with the response variable and therefore included in the model.

5. The Wald test for each level of the categorical variable is also shown. This tests the difference between that level and the baseline category.

##### Propensity score matching

Propensity score matching (PSM) is a method that allows cases from a treatment sample (in this case the HSE Core sample in London) to be matched to cases from a control sample (the London Boost Sample). The matching controls for differences in sample profile; in this case the socio-demographic profile of the Core sample is adjusted to make it match that of the Boost. Matching the samples means any differences in survey estimates should be attributable to measurement error and not sample composition.

PSM is based on the following steps:

1. A propensity model is fitted using logistic regression, the binary outcome variable is whether the case belongs to the treatment or control sample;
2. A predicted score is generated by the model for each case, this is the 'propensity score';
3. The propensity scores are then used to match the treatment and control samples[[3]](#footnote-4). The matched samples are then analysed together.

The first step was to model the differences between the two sample profiles. The probability, or propensity, of the participant being in either the Core or Boost sample was estimated using a logistic regression model. Sample type was used as the dependent variable and a number of socio-demographic characteristics were used as predictors. The predicted probabilities were saved as propensity scores. These scores measure the propensity of a participant to be in either the Core or Boost sample, depending on their socio-demographic characteristics. The propensity scores were used to match the samples, since participants with similar scores should be similar in terms of the characteristics in the model. A full list of the demographic and household characteristics used in the regression model is given in Table B1.

Table B1 Variables included in the logistic regression

|  |
| --- |
| Age group |
| Sex |
| Ethnicity |
| Marital status |
| NS-Sec of HRP |
| Current economic activity of HRP |
| Number of adults in the household (16+) |
| Number of children in the household (0-15) |
| Household type |
| Lone parent household |
| Index of Multiple Deprivation score 2006 (Super Output Area level) |
| PCT spearhead status |
| % of the population from a non-white background (Postcode sector level measure using data from 2001 Census) |
| % of the population from a non-manual occupation (Postcode sector level measure using data from 2001 Census) |

There were some variables that were unsuitable for using in the model. These were household income and the participant’s current economic activity and education.

These variables were excluded because there were fundamental differences in the wording or format of the Core and Boost questions, which meant that it was unclear whether the response categories were measuring the same thing. These variables also contained large numbers of missing cases for Boost participants

Men and women were matched separately. For women the variables that varied significantly by sample type were NS-SEC of HRP, participant ethnicity and IMD score of the local area. For men the significant variables were household type, NS-SEC of HRP, participant ethnicity and IMD score of the local area.

Women in the Core sample were more likely to have a HRP in an intermediate NS-SEC category than Boost participants, who were more likely to have a HRP at either end of the NS-SEC scale (either higher managers or had never worked). Women in the Boost were more likely to be from a White ethnic background and were more likely to live in more deprived areas, since being in the Boost sample was associated with a higher IMD score.

Similar patterns were seen for men. Core participants were more likely to have a HRP in a middle NS-SEC category. Boost participants were more likely to have a HRP who was either in management or had never worked. Men in the Boost participants were also more likely to be from a White ethnic background and live in more deprived areas. The full models are given in Tables B2 and B3, below.

Table B2 Propensity model for women

|  | **B** | **S.E.** | **Wald** | **df** | **Sig.** | **Exp(B)** |
| --- | --- | --- | --- | --- | --- | --- |
|  |  |  |  |  |  |  |
| NS-SEC of HRP (8 variable classification) | | | 16.8 | 7 | 0.019 |  |
| Higher managerial and professional occupations |  |  | (baseline) |  |  | 1.00 |
| Lower managerial and professional occupations | -0.22 | 0.13 | 3.0 | 1 | 0.082 | 0.80 |
| Intermediate occupations | -0.09 | 0.16 | 0.3 | 1 | 0.596 | 0.92 |
| Small employers and own account workers | -0.23 | 0.17 | 1.8 | 1 | 0.180 | 0.79 |
| Lower supervisory and technical occupations | -0.33 | 0.19 | 3.1 | 1 | 0.080 | 0.72 |
| Semi-routine occupations | -0.46 | 0.15 | 9.3 | 1 | 0.002 | 0.63 |
| Routine occupations | -0.38 | 0.17 | 5.0 | 1 | 0.025 | 0.68 |
| Never worked and long term unemployed | 0.11 | 0.19 | 0.3 | 1 | 0.577 | 1.11 |
|  |  |  |  |  |  |  |
| Ethnicity of participants | | | 24.7 | 4 | 0.000 |  |
| White |  |  | (baseline) |  |  | 1.00 |
| Mixed | -0.05 | 0.25 | 0.0 | 1 | 0.830 | 0.95 |
| Asian | -0.39 | 0.12 | 11.3 | 1 | 0.001 | 0.68 |
| Black | -0.55 | 0.13 | 17.8 | 1 | 0.000 | 0.58 |
| Chinese/other | -0.19 | 0.21 | 0.8 | 1 | 0.365 | 0.82 |
|  |  |  |  |  |  |  |
| Age group |  |  | 2.0 | 5 | 0.851 |  |
| 16-24 |  |  | (baseline) |  |  | 1.00 |
| 25-34 | -0.10 | 0.14 | 0.5 | 1 | 0.489 | 0.91 |
| 35-44 | -0.16 | 0.14 | 1.3 | 1 | 0.262 | 0.85 |
| 45-54 | -0.05 | 0.15 | 0.1 | 1 | 0.725 | 0.95 |
| 55-64 | -0.01 | 0.17 | 0.0 | 1 | 0.958 | 0.99 |
| 65+ | -0.11 | 0.15 | 0.5 | 1 | 0.463 | 0.89 |
|  |  |  |  |  |  |  |
| IMD score | 0.02 | 0.00 | 24.6 | 1 | 0.000 | 1.02 |
|  |  |  |  |  |  |  |
| Constant | 1.22 | 0.17 | 53.6 | 1 | 0.000 | 3.39 |

Notes: 1. The response is 1 = Boost, 0 = Core.

2. The model R^2^ = 0.019 (Cox and Snells).

3. B is the estimate coefficient with standard error S.E.

4. The Wald-test measures the impact of the categorical variable on the model with the appropriate number of degrees of freedom df. If the test is significant (sig < 0.05) then the categorical variable is considered to be ‘significantly associated’ with the response variable and therefore included in the model.

5. The Wald test for each level of the categorical variable is also shown. This tests the difference between that level and the baseline category.

Table B3 Propensity model for men

|  | **B** | **S.E.** | **Wald** | **df** | **Sig.** | **Exp(B)** |
| --- | --- | --- | --- | --- | --- | --- |
|  |  |  |  |  |  |  |
| Household type |  |  | 18.0 | 6 | 0.006 |  |
| 1 adult aged 16-59, no children |  |  | (baseline) |  |  | 1.00 |
| 2 adults, both 16-59, no children | 0.19 | 0.16 | 1.4 | 1 | 0.235 | 1.21 |
| Small family | 0.40 | 0.17 | 5.6 | 1 | 0.018 | 1.49 |
| Large family | 0.21 | 0.20 | 1.1 | 1 | 0.301 | 1.23 |
| Large adult household | 0.58 | 0.16 | 13.2 | 1 | 0.000 | 1.78 |
| 2 adults, 1 or both aged 60+, no children | 0.34 | 0.22 | 2.4 | 1 | 0.118 | 1.41 |
| 1 adult, aged 60+, no children | 0.12 | 0.26 | 0.2 | 1 | 0.657 | 1.12 |
|  |  |  |  |  |  |  |
| NS-SEC of HRP (8 variable classification) | |  | 17.4 | 7 | 0.015 |  |
| Higher managerial and professional occupations |  |  | (baseline) |  |  |  |
| Lower managerial and professional occupations | 0.00 | 0.13 | 0.0 | 1 | 0.993 | 1.00 |
| Intermediate occupations | -0.10 | 0.18 | 0.3 | 1 | 0.578 | 0.90 |
| Small employers and own account workers | -0.10 | 0.17 | 0.4 | 1 | 0.552 | 0.90 |
| Lower supervisory and technical occupations | -0.38 | 0.17 | 4.6 | 1 | 0.031 | 0.69 |
| Semi-routine occupations | -0.34 | 0.17 | 4.2 | 1 | 0.041 | 0.71 |
| Routine occupations | -0.37 | 0.17 | 4.8 | 1 | 0.028 | 0.69 |
| Never worked and long term unemployed | 0.35 | 0.24 | 2.1 | 1 | 0.147 | 1.41 |
|  |  |  |  |  |  |  |
| Ethnicity of participants |  |  | 20.6 | 4 | 0.000 |  |
| White |  |  | (baseline) |  |  | 1.00 |
| Mixed | 0.37 | 0.33 | 1.3 | 1 | 0.263 | 1.44 |
| Asian | -0.30 | 0.13 | 5.6 | 1 | 0.018 | 0.74 |
| Black | -0.56 | 0.15 | 14.8 | 1 | 0.000 | 0.57 |
| Chinese/other | 0.12 | 0.25 | 0.2 | 1 | 0.647 | 1.12 |
|  |  |  |  |  |  |  |
| Age group |  |  | 3.9 | 5 | 0.569 |  |
| 16-24 |  |  | (baseline) |  |  | 1.00 |
| 25-34 | 0.04 | 0.16 | 0.1 | 1 | 0.803 | 1.04 |
| 35-44 | 0.19 | 0.17 | 1.3 | 1 | 0.255 | 1.21 |
| 45-54 | 0.26 | 0.17 | 2.4 | 1 | 0.123 | 1.30 |
| 55-64 | 0.15 | 0.19 | 0.7 | 1 | 0.417 | 1.17 |
| 65+ | 0.18 | 0.22 | 0.6 | 1 | 0.423 | 1.20 |
|  |  |  |  |  |  |  |
| IMD score | 0.01 | 0.00 | 10.4 | 1 | 0.001 | 1.01 |
|  |  |  |  |  |  |  |
| Constant | 0.57 | 0.22 | 6.6 | 1 | 0.010 | 1.78 |

Notes: 1. The response is 1 = Boost, 0 = Core.

2. The model R^2^ = 0.017 (Cox and Snells).

3. B is the estimate coefficient with standard error S.E.

4. The Wald-test measures the impact of the categorical variable on the model with the appropriate number of degrees of freedom df. If the test is significant (sig < 0.05) then the categorical variable is considered to be ‘significantly associated’ with the response variable and therefore included in the model.

5. The Wald test for each level of the categorical variable is also shown. This tests the difference between that level and the baseline category.

The two samples are fairly similar in size; this means one-to-one matching would have been unsuitable. Propensity score matching usually requires a large group of controls to find suitable one-on-one matches for a smaller treatment group. It would have been difficult to carry out one-to-one matching (without replacement) between the Boost and Core samples, and the kernel method of matching was used instead

The kernel function matches each participant in the Boost to *all* the participants in the Core. The participants in the Boost that are matched are weighted according to their proximity to each Core participant using their propensity score. The kernel function weights the contribution of each participant in the Core sample, with higher weights for participants who are a better match. Exact matches get a large weight; poorer matches contribute less and get a smaller weight. All members of the Core are used but the poorer matches have such small weights they have little effect.

The output of the matching process is a weight which, when applied to the Core data, makes the Core match the Boost sample in terms of the socio-demographic variables in the model. The participants in the Boost sample are each given a weight of 1. The weights for the Core sample vary and ensure that the profiles of the two groups match. The Core sample was adjusted to match the unweighted Boost sample, so neither sample is representative of the population of London and cannot be used to make generalisations. The samples should only be compared to each other, and not to the London population. When running analyses on matched combined data we can attribute any differences in the outcome variables to the survey mode and not to differences in sample composition.

1. PSUs were postcode sectors or groups of postcode sectors. Postcode sectors with fewer than 500 addresses were grouped with neighbouring sectors. [↑](#footnote-ref-2)
2. These were the most recent estimates available when the HSE was being weighted. For the sake of comparison the same estimates were subsequently used for the Boost. [↑](#footnote-ref-3)
3. The propensity score matching was carried out in Stata V9 using the psmatch2 command [↑](#footnote-ref-4)
